# Forced-Vibration Characteristics of Bowtie-Shaped Honeycomb Composite Sandwich Panel with Viscoelastic Damping Layer

**DOI:** 10.3390/ma17164067

**Published:** 2024-08-16

**Authors:** Siqi Miao, Yifeng Zhong, Mingtao Zhang, Rong Liu

**Affiliations:** 1 School of Civil Engineering, Chongqing University, Chongqing 400045, China; 18990932778@163.com (S.M.); 18782485584@163.com (M.Z.); 202016131332@cqu.edu.cn (R.L.); 2 Key Laboratory of New Technology for Construction of Cities in Mountain Area, Chongqing University, Chongqing 400045, China

**Keywords:** forced-vibration characteristics, bowtie-shaped honeycomb, composite sandwich panel, auxetic effect, variational asymptotic method

## Abstract

The incorporation of viscoelastic layers in laminates can markedly enhance the damped dynamic characteristics. This study focuses on integrating viscoelastic layers into the composite facesheet of the bowtie-shaped honeycomb core composite sandwich panel (BHC-CSP). The homogenization of the damped BHC-CSP is performed by employing the variational asymptotic method. Based on the generalized total energy equation, the energy functional of the representative unit cell of the damped BHC-CSP is asymptotically analyzed. The warping function, derived following the principle of minimum potential energy, provides a basis for obtaining the corresponding Euler–Lagrange equation to ascertain the equivalent elastic properties of the damped BHC-CSP. Utilizing the developed two-dimensional equivalent model, the free-vibration characteristics of the damped BHC-CSP are examined across diverse boundary conditions while delving into the impact of an external viscous damping layer on the natural frequency of the damped BHC-CSP. The results reveal that intensified boundary constraints effectively diminish the effective vibration region of the damped BHC-CSP, thereby enhancing its overall stability. The introduction of a PMI foam layer proves effective in adjusting the stiffness and mass distribution of the damped BHC-CSP. Resonance characteristics are explored through frequency and time-domain analyses, highlighting the pivotal roles of the excitation position and receiver point in influencing the displacement and velocity responses. Although the stiffness is improved by incorporating a PMI foam layer, its effect on the damping performance of the damped BHC-CSP is minimal when compared to the T-SW308 foam layer.

## 1. Introduction

The mechanic performance of sandwich structures is significantly influenced by both the structural configuration and the core material [1,2]. In recent years, there have been notable advancements in honeycomb sandwich panel design, including more intricate core shapes and innovative combinations with high-performance composite facesheets. Among different types of honeycomb sandwich panels, the re-entrant hexagonal honeycomb sandwich panel is notable for possessing a negative Poisson’s ratio, displaying unconventional behavior under loading, expanding under tension, and contracting under compression [3,4]. This unique mechanical response enhances the deformability and flexibility, leading to improved energy absorption and dissipation capabilities. Consequently, it exhibits an exceptional performance in vibration damping and impact resistance [5,6].

Numerous works have been dedicated to exploring the vibration characteristics of honeycomb sandwich panels. Jiang et al. [7] explored the free-vibration characteristics of composite sandwich plates with a re-entrant honeycomb core using theory, experiments, and the FE method. Sun et al. [8] conducted numerical and experimental investigations on the impact damage of aluminum honeycomb sandwich panels, focusing on the effect of interface layer geometry. Zhang et al. [9] explored the low-speed impact behavior of CFRP composite panels and Nomex honeycomb sandwich panels using numerical and experimental methods. Li et al. [10] investigated the anti-knock performance of hexagonal aluminum honeycomb sandwich panels through explosion testing. Current research on the dynamic performance of honeycomb sandwich panels primarily concentrates on specific structural parameters, material combinations, or restricted impact conditions, thereby constraining the generalizability and universality of research findings.

**Figure 1:**
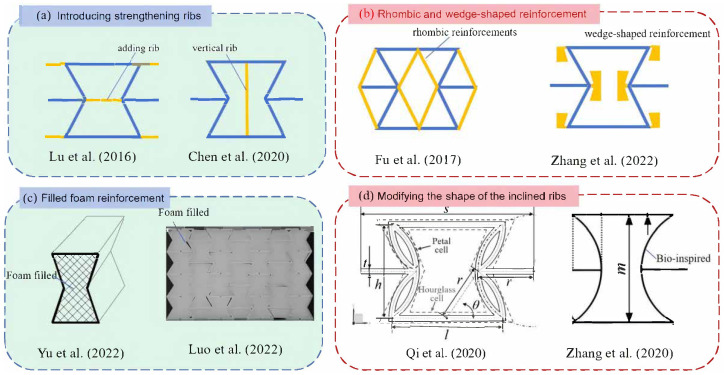
Recent studies focusing on enhancing the stiffness of re-entrant honeycomb structures (the yellow ribs serve as additional stiffeners): (**a**) introducing strengthening ribs [11,12], (**b**) rhombic [13] and wedge-shaped reinforcement [14], (**c**) filled foam reinforcement [15,16], and (**d**) modifying the shape of the inclined ribs [17,18].

Re-entrant honeycombs typically exhibit high porosity with a relatively low effective stiffness, thereby restricting their practical applications in fields demanding high stiffness. To enhance the stiffness of regular re-entrant honeycombs, numerous novel variants have been devised, as illustrated in Figure 1. For instance, Lu et al. [11] introduced narrow ribs within the re-entrant cellular structure, observing a clear linear correlation between Poisson’s ratio and Young’s modulus with varying rib thickness. Chen et al. [12] incorporated strengthening ribs into traditional auxetic unit cells perpendicular to the re-entrant direction and noted a substantial enhancement of Young’s modulus by approximately 200% in the strengthening direction without significant compromise to auxecitical properties.

Fu et al. [13] introduced a novel honeycomb design by incorporating a rhombic pattern into the traditional re-entrant hexagonal honeycomb structure to improve its in-plane mechanical properties. Zhang et al. [14] created a novel re-entrant honeycomb structure by incorporating wedge-shaped components into the traditional design. Yu et al. [15] studied the mechanical properties of a novel foam-filled 2D re-entrant hexagonal honeycomb structure subjected to planar compression. Luo et al. [16] filled slow recovery foam into re-entrant honeycombs to enhance its mechanical characteristics. Qi et al. [17] introduced a new re-entrant circular honeycomb design that features double-circle arc cell walls instead of sloped walls. Zhang et al. [18] introduced a re-entrant arc-shaped honeycomb model based on the bio-inspired structural design.

**Figure 2:**
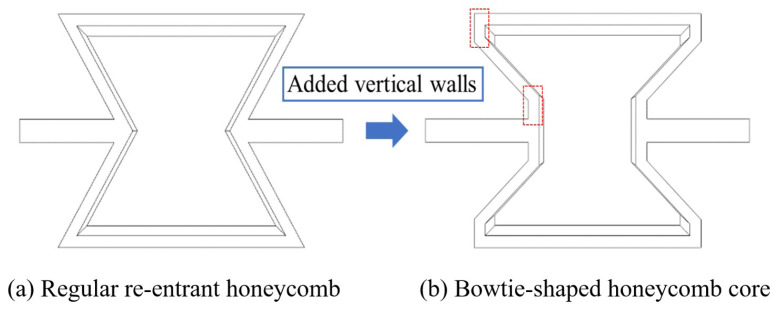
Regularre-entrant honeycomb and its variant bowtie-shaped honeycomb achieved by adding vertical walls highlighted in red boxes in (**b**).

Inspired by recent advancements in the field, a novel composite sandwich panel, called the bowtie-shaped honeycomb core composite sandwich panel (BHC-CSP), has been developed by integrating vertical walls into the core layer of a re-entrant hexagonal honeycomb sandwich panel, as depicted in Figure 2. This modification enhances the contact area between adjacent cells and enhances the connections within the cellular structure, leading to an improved overall performance. Nonetheless, enhancing the structural damping characteristics is imperative to attain efficient vibration mitigation and noise management in line with engineering specifications. Inserting viscoelastic layers in laminates can significantly improve the damped dynamic properties. Moreover, the concept of interlaminar damping is also well-suited to the manufacturing processes involved in creating laminated structures.

The literature offers limited studies on the vibration and damping characteristics of composite laminates with viscoelastic layers [19,20,21,22]. Cupial et al. [20] applied the first-order shear deformation theory to evaluate the loss factors of three-layered rectangular plates with a viscoelastic core layer and laminated facesheets. Plagianakos et al. [22] presented a higher-order theory and utilized finite element analysis to assess the modal damping of laminates with ply-angle variations and interply damping layers. Yang et al. [23] investigated the vibration and damping characteristics of hybrid carbon fiber composite pyramidal truss sandwich panels with viscoelastic layers integrated into the facesheets. Jean et al. [24] investigated the damping characteristics of unidirectional glass fiber composites with interleaved viscoelastic layers. The results indicate that hybrid composite sandwich structures with viscoelastic layers provide an effective method for improving damping performance.

Due to its innovation and potential applications, a multiscale study on the vibration and damping characteristics of the BHC-CSP is essential. Zhong and Yu [25,26,27] introduced a novel multiscale modeling approach for composite structures utilizing the variational asymptotic method (VAM) developed by Hodges and Cesnik [28]. On the microscopic scale, the effective properties of composite structures are obtained through asymptotic homogenization analysis and assigned to equivalent models. The strain energy of representative cells is obtained through homogenization analysis by applying the principle of minimum total potential energy, and the necessary performance parameters for macroscopic structure calculation (such as equivalent stiffness) are derived. Subsequently, the stress and strain responses of the macroscopic scale structure are obtained through macroscopic analysis. The introduction of a small parameter (such as the width-to-thickness ratio) allows for the gradual expansion of the energy functional, achieving a balance between calculation accuracy and efficiency [29,30,31].

To the best of our knowledge, the forced-vibration characteristics of the BHC-CSP, considering the damping layer, have not been reported. Therefore, in this work, a more precise and reliable VAM-based model is established to analyze the vibration characteristics of the damped BHC-CSP. The paper proceeds as follows: Section 2 and Section 3 present the theoretical formula and constitutive relationship of the two-dimensional equivalent Reissner-Mindlin model (2D-ERM) using the VAM. In Section 4, the forced-vibration analysis of the 2D-ERM considering a viscoelastic damping layer is conducted. Section 5 validates the effectiveness and precision of the 2D-ERM in analyses of free and forced vibrations of the damped BHC-CSP under different cases. In Section 6, the impact of geometric parameters on the vibration characteristics of the damped BHC-CSP is investigated. Section 7 compares the vibration characteristics of various damped honeycomb sandwich panels. Finally, Section 8 summarizes the primary conclusions of the study.

## 2. VAM-Based Homogenization of Composite Facesheets with Viscous Damping Layer

**Figure 3:**
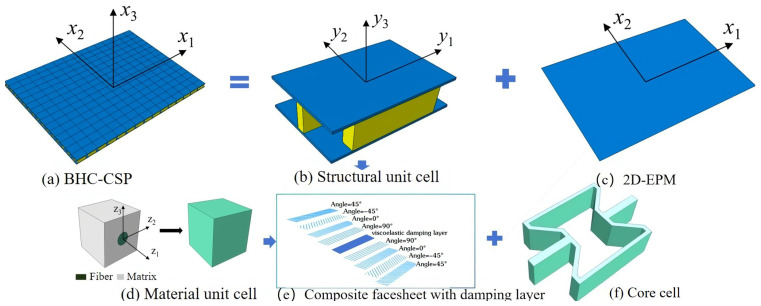
Schematic diagram of equivalent analysis of bowtie-shaped honeycomb core composite sandwich panel (BHC-CSP).

First, the VAM is employed to homogenize the laminated facesheet with the viscous damping layer, as shown in Figure 3d. The material kinematics are characterized by both the volume-averaged value and the corresponding difference:

(1) $$u_{i}\left(y;z\right)=v_{i}\left(y\right)+\eta w_{i}\left(y;z\right),$$

where $u_{i}$ and $v_{i}$ represent the displacements of the heterogeneous and homogeneous materials, respectively. $y=\left(y_{1},y_{2},y_{3}\right)$ and $z=\left(z_{1},z_{2},z_{3}\right)$ represent the meso- and microcoordinate systems, respectively. $w_{i}$ is the difference function signifying the variance between $u_{i}$ and $v_{i}$ (i,j,k,l=1,2,3, and repeated subscripts imply summation).

The strain field within the heterogeneous material can be expressed as

(2) $$\varepsilon_{ij}\left(y;z\right)=\frac{1}{2}\left[\frac{\partial u_{i}\left(y;z\right)}{\partial y_{j}}+\frac{\partial u_{j}\left(y;z\right)}{\partial y_{i}}\right]={\bar{\varepsilon }}_{ij}+\eta w_{i|j},$$

where ${\bar{\varepsilon }}_{ij}=\frac{1}{2}\left(\frac{\partial v_{i}}{\partial y_{j}}+\frac{\partial v_{j}}{\partial y_{i}}\right)$ represents the strain field of the homogeneous material, $w_{i|j}=\frac{1}{2}\left(\frac{\partial w_{i}}{\partial z_{j}}+\frac{\partial w_{j}}{\partial z_{i}}\right)$.

The kinematic variables of homogeneous materials can be expressed as

(3) $$v_{i}=\frac{1}{\Omega }\int_{\Omega }u_{i}\;d\Omega \equiv \langle u_{i}\rangle ,{\bar{\varepsilon }}_{\mathrm{ij}}=\langle \varepsilon_{\mathrm{ij}}\rangle ,$$

where 〈·〉 represents the integration over the volume domain of the unit cell.

The constraint on the difference function can be obtained from Equation (3), such as

(4) $$\langle w_{i}\rangle =0,\;\langle w_{i|j}\rangle =0,$$

The energy difference between the deformed inhomogeneous material and the homogeneous material can be specified as

(5) $$\delta J=\delta \left(\Pi_{\mathrm{micro}\;}-\Pi_{\mathrm{macro}\;}-\lambda_{kl}\langle w_{k|l}\rangle -\lambda_{i}\langle w_{i}\rangle \right)=0,$$

where $\Pi_{\mathrm{micro}\;}=\langle C_{ijkl}\varepsilon_{ij}\varepsilon_{kl}\rangle =\langle C_{ijkl}\left({\bar{\varepsilon }}_{ij}+w_{i|j}\right)\left({\bar{\varepsilon }}_{kl}+w_{kl}\right)\rangle ;\Pi_{\mathrm{macro}\;}=\langle {\bar{C}}_{ijkl}{\bar{c}}_{ij}{\bar{\varepsilon }}_{kl}\rangle$, $C_{ijkl}$ is the fourth-order elastic tensor; and $\lambda_{kl}$ and $\lambda_{i}$ are the Lagrange multipliers to introduce the constraints in Equation (4).

By minimizing the energy difference subject to the constraints in Equation (4), one obtains

(6) $$\min_{w_{i}\in \mathrm{Eq}\cdot \left(4\right)}\langle \frac{1}{2}C_{ijkl}\varepsilon_{ij}\varepsilon_{kl}\rangle =\min_{w_{i}\in \mathrm{Eq}\cdot \left(4\right)}\langle \frac{1}{2}C_{ijkl}\left({\bar{\varepsilon }}_{ij}+w_{i|j}\right)\left({\bar{\varepsilon }}_{kl}+w_{k|l}\right)\rangle ,$$

The variational expression can be formulated as

(7) $$U=\frac{1}{2}\langle {\left(\Gamma_{h}w+\bar{\epsilon }\right)}^{T}C\left(\Gamma_{h}w+\bar{\epsilon }\right)\rangle ,$$

where C is the 6×6 material matrix and $w={\left[w_{1},w_{2},w_{3}\right]}^{T}$ is the global strain array; $\bar{\epsilon }={\left[\begin{array}{cccccc} {\bar{\varepsilon }}_{11} & {\bar{\varepsilon }}_{22} & {\bar{\varepsilon }}_{33} & 2{\bar{\varepsilon }}_{23} & 2{\bar{\varepsilon }}_{13} & 2{\bar{\varepsilon }}_{12} \end{array}\right]}^{T}$, $\Gamma_{h}={\left[\begin{array}{cccccc} \partial /\partial z_{1} & 0 & 0 & 0 & \partial /\partial z_{3} & \partial /\partial z_{2}\\ 0 & \partial /\partial z_{2} & 0 & \partial /\partial z_{3} & 0 & \partial /\partial z_{3}\\ 0 & 0 & \partial /\partial z_{3} & \partial /\partial z_{2} & \partial /\partial z_{1} & 0 \end{array}\right]}^{T}$.

The discrepancy function can be discretized utilizing shape functions that are defined on the unit cell:

(8) $$w\left(y_{i};z_{i}\right)=S\left(z_{i}\right)N\left(y_{i}\right),$$

where the shape function *S* depends on the element type employed and *N* represents the nodal values of the difference function that needs to be solved.

Substituting Equation (8) into Equation (7), one can express the discrete form of the strain energy functional as

(9) $$U=\frac{1}{2}\left(N^{T}D_{\mathrm{hh}}N+2N^{T}D_{h\varepsilon }\bar{e}+{\bar{\epsilon }}^{T}D_{\varepsilon \varepsilon }\bar{\bar{\varepsilon }}\right)-\frac{1}{2}N^{T}\bar{D}N-\lambda D_{h\lambda }^{T}N,$$

where

(10) $$D_{\mathrm{hh}}=\langle {\left(\Gamma_{h}S\right)}^{T}C\left(\Gamma_{h}S\right)\rangle ,D_{he}=\langle {\left(\Gamma_{h}S\right)}^{T}C\rangle ,D_{ce}=\langle C\rangle ,D_{h\lambda }={\langle \Gamma_{h}S\rangle }^{T}.$$

Minimizing Equation (9) leads to the linear system

(11) $$D_{\mathrm{hh}}N=-D_{hs}\bar{\epsilon }.$$

It is evident that *N* is directly proportional to ϵ, and the solution can be expressed as

(12) $$N=N_{0}\bar{\epsilon },\;w=SN_{0}\bar{\epsilon }.$$

By substituting Equation (12) into Equation (9), one can calculate the energy stored in the unit cell as

(13) $$U=\frac{1}{2}{\bar{\epsilon }}^{T}\left(2N_{0}^{T}D_{h\varepsilon }+N_{0}^{T}D_{hh}N_{0}+D_{\varepsilon E}\right)\bar{\epsilon }=\frac{\Omega }{2}{\bar{\epsilon }}^{T}\bar{C}\bar{\epsilon },$$

where $\bar{C}$ is the equivalent material matrix.

## 3. VAM-Based Equivalent Reissner-Mindlin Model for BHC-CSP

The process of establishing the 2D-ERM of the BHC-CSP using the VAM is depicted in Figure 3b. The VAM-based equivalent Reissner-Mindlin model involves representing the displacement field ($u_{i}$) of the BHC-CSP using the displacements (${\bar{u}}_{i}$) in the 2D-ERM and warping functions $w_{i}$, such as

(14) $$\begin{array}{cc} \; & u_{1}\left(x_{1},x_{2},y_{1},y_{2},y_{3},t\right)={\bar{u}}_{1}\left(x_{1},x_{2},t\right)-\zeta y_{3}{\bar{u}}_{3,1}\left(x_{1},x_{2},t\right)+\zeta w_{1}\left(x_{1},x_{2},y_{1},y_{2},y_{3},t\right),\\ \; & u_{2}\left(x_{1},x_{2},y_{1},y_{2},y_{3},t\right)={\bar{u}}_{2}\left(x_{1},x_{2},t\right)-\zeta y_{3}{\bar{u}}_{3,2}\left(x_{1},x_{2},t\right)+\zeta w_{2}\left(x_{1},x_{2},y_{1},y_{2},y_{3},t\right),\\ \; & {\bar{u}}_{3}\left(x_{1},x_{2},y_{1},y_{2},y_{3},t\right)={\bar{u}}_{3}\left(x_{1},x_{2},t\right)+\zeta w_{3}\left(x_{1},x_{2},y_{1},y_{2},y_{3},t\right). \end{array}$$

The explicit representation of ${\bar{u}}_{i}$ can be derived from Equation (14),

(15) $${\bar{u}}_{1}=\langle u_{1}\rangle +\zeta \langle y_{3}\rangle {\bar{u}}_{3,1},\;{\bar{u}}_{2}=\langle u_{2}\rangle +\zeta \langle y_{3}\rangle {\bar{u}}_{3,2},\;{\bar{u}}_{3}=\langle u_{3}\rangle ,$$

where 〈·〉 represents the volume average over the unit cell.

Since the meso-coordinate $y_{i}$ originates at the geometric center of the unit cell, it follows that $\langle y_{3}\rangle =0$. This results in three constraints on the warping functions:

(16) $$\langle w_{i}\rangle =0.$$

The concept of rotation tensor decomposition can be used to express the 3D strain components with small local rotation as

(17) $$\varepsilon_{ij}=\frac{1}{2}\left(\frac{\partial u_{i}}{\partial x_{j}}+\frac{\partial u_{j}}{\partial x_{i}}\right).$$

The 3D strain field Γ can be represented in matrix form as

(18) $$\begin{array}{c} \Gamma_{e}={\left[\varepsilon_{11}\;\varepsilon_{22}\;2\varepsilon_{12}\right]}^{T}=\epsilon +\zeta y_{3}\kappa +I_{\alpha }w_{∥,\alpha },\\ 2\Gamma_{s}={\left[2\varepsilon_{13}\;2\varepsilon_{23}\right]}^{T}=w_{∥,3}+e_{\alpha }w_{3,\alpha },\\ \Gamma_{t}=\varepsilon_{33}=w_{3,3}, \end{array}$$

where ${\left(\right)}_{\left|\right|}={\left[{\left(\right)}_{1}\;{\left(\right)}_{2}\right]}^{T}$, $\epsilon ={\left[\begin{array}{ccc} \epsilon_{11} & 2\epsilon_{12} & \epsilon_{22} \end{array}\right]}^{T}$, $\kappa ={\left[\kappa_{11}\;\kappa_{12}+\kappa_{21}\;\kappa_{22}\right]}^{T}$, and

(19) $$I_{1}=\left[\begin{array}{cc} 1 & 0\\ 0 & 1\\ 0 & 0 \end{array}\right],\;I_{2}=\left[\begin{array}{cc} 0 & 0\\ 1 & 0\\ 0 & 1 \end{array}\right],\;e_{1}=\left\{\begin{array}{c} 1\\ 0 \end{array}\right\},\;e_{2}=\left\{\begin{array}{c} 0\\ 1 \end{array}\right\}.$$

The panel’s stored strain energy can be represented as

(20) $$U={\;\int \int \;}_{s}\frac{1}{\Omega }U_{\Omega }dx_{1}dx_{2},$$

where *s* is the reference surface, with an integral domain of $-a/2<x_{1}<a/2,-b/2<x_{2}<b/2$, where *a* and *b* are the length and width of the sandwich panel, respectively; $U_{\Omega }/\Omega$ represents the strain energy density.

**Figure 4:**
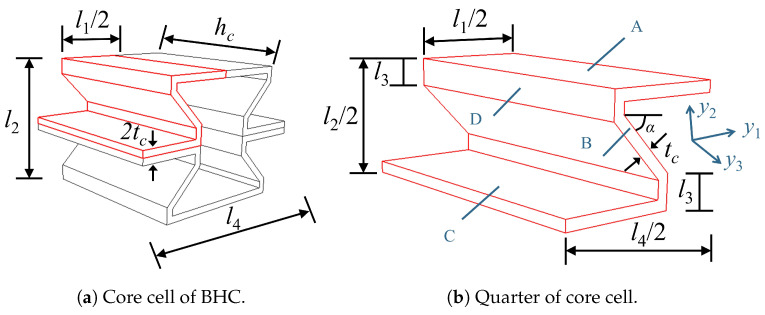
Core cell decomposition for strain energy integral.

Based on the principle of conservation energy, the strain energy is equivalent to the total energy stored by all its components:

(21) $$\begin{array}{cc} U_{\Omega }= & {\;\int \int \int \;}_{V_{b}}\Gamma_{b}^{T}D_{b}\Gamma_{b}dy_{1}dy_{2}dy_{3}+{\;\int \int \int \;}_{V_{t}}\Gamma_{t}^{T}D_{t}\Gamma_{t}dy_{1}dy_{2}dy_{3}\\ \; & +4\times \left({\;\int \int \int \;}_{V_{A}}\Gamma_{A}^{T}D_{A}\Gamma_{A}dy_{1}dy_{2}dy_{3}+{\;\int \int \int \;}_{V_{B}}\Gamma_{B}^{T}D_{B}\Gamma_{B}dy_{1}dy_{2}dy_{3}\right.\\ \; & \left.+{\;\int \int \int \;}_{V_{c}}\Gamma_{C}^{T}D_{C}\Gamma_{C}dy_{1}dy_{2}dy_{3}+2\times {\;\int \int \int \;}_{V_{D}}\Gamma_{D}^{T}D_{D}\Gamma_{D}dy_{1}dy_{2}dy_{3}\right) \end{array},$$

where subscripts *b* and *t* represent the bottom and top facesheets, respectively; A,B,C, and *D* represent different parts within the 1/4 core, as shown in Figure 4.

Since the coordinates originate from the geometric center of the core cell, the integration domains of the top and bottom facesheets in Equation (21) are identical, with the exception of $V_{b},-h_{c}/2<y_{3}<-h_{c}/2-h_{f}$ and $V_{t},h_{c}/2<y_{3}<h_{c}/2+h_{f}$. In the same way, the integral domain of the core layer is the same along the $y_{3}$ axis, and the differences are

(22) $$\begin{array}{c} V_{A},0<y_{1}<-l_{1}/2+t_{c},\;l_{2}/2-t_{c}<y_{2}<l_{2}/2\\ V_{B},-l_{1}/2+\left(l_{2}/2-l_{3}\right)/\tan\alpha <y_{1}<-l_{1}/2,\;l_{3}<y_{2}<l_{2}/2-l_{3}\\ V_{C},-l_{1}/2+\left(l_{2}/2-l_{3}\right)/\tan\alpha -t_{c}<y_{1}<-l_{4}/2,\;0<y_{2}<t_{c}\\ V_{D},-l_{1}/2+t_{c}<y_{1}<-l_{1}/2,\;l_{2}/2-l_{3}<y_{2}<l_{2}/2 \end{array}$$

Equation (21) can be written as

(23) $$U=\frac{1}{2}\langle \Gamma^{T}D\Gamma \rangle =\frac{1}{2}\langle {\left\{\begin{array}{c} \Gamma_{e}\\ 2\Gamma_{s}\\ \Gamma_{t} \end{array}\right\}}^{T}\left[\begin{array}{ccc} C_{e} & C_{es} & C_{et}\\ C_{es}^{T} & C_{s} & C_{st}\\ C_{et}^{T} & C_{st}^{T} & C_{t} \end{array}\right]\left\{\begin{array}{c} \Gamma_{e}\\ 2\Gamma_{s}\\ \Gamma_{t} \end{array}\right\}\rangle ,$$

where $C_{e},C_{es},C_{et},C_{s},C_{st}$, and $C_{t}$ represent the respective submatrices of the 3D 6×6 material matrix.

The kinetic energy of the BHC-CSP can be obtained by

(24) $$K=\frac{1}{2}\int_{V}\rho v^{T}vdV=K_{2D}+K^{*},$$

where ρ denotes the mass density, *v* represents the absolute velocity of a generic point in the panel, and

(25) $$K_{2D}=\frac{1}{2}\int_{\Omega }\left(\bar{\mu }V^{T}V+2\omega^{T}\tilde{\mu \bar{\xi }}V+\omega^{T}j\omega \right)d\Omega ,$$

(26) $$K^{*}=\frac{1}{2}\int_{V}\rho \left[{\left(\tilde{\omega }w+\dot{w}\right)}^{T}\left(\tilde{\omega }w+\dot{w}\right)+2{\left(V+\tilde{\omega }\xi \right)}^{T}\left(\tilde{\omega }w+\dot{w}\right)\right]dV$$

where $\bar{\mu },\mu \bar{\xi }$, and *j* are inertial constants commonly used in plate dynamics.

The virtual work resulting from applied loads can be calculated as

(27) $$\bar{\delta W}={\bar{\delta W}}_{2D}+{\bar{\delta W}}^{*},$$

where

(28) $${\bar{\delta W}}_{2D}=\int_{s}\left(f_{i}{\bar{\delta q}}_{i}+m_{\alpha }{\bar{\delta \psi }}_{\alpha }\right)ds+\int_{\partial s}\left(\langle Q_{i}\rangle {\bar{\delta q}}_{i}+\langle x_{3}Q_{\alpha }\rangle {\bar{\delta \psi }}_{\alpha }\right)ds,$$

(29) $${\bar{\delta W}}^{*}=\int_{s}\left(\langle P_{i}\delta w_{i}\rangle +\tau_{i}\delta w_{i}^{+}+\beta_{i}\delta w_{i}^{-}\right)ds+\int_{\partial s}\langle Q_{i}\delta w_{i}\rangle ds,$$

with $m_{\alpha }$ and $f_{i}$ denoting the generalized moments and forces, respectively; ${\bar{\delta \psi }}_{i}$ and ${\bar{\delta q}}_{i}$ are virtual rotation and displacement, respectively.

For simplicity, the virtual work conducted by the warping function along the lateral boundaries of the panel can be neglected to derive the extended Hamilton’s principle as

(30) $$\int_{t_{1}}^{t_{2}}\left[\delta \left(K_{2D}+K^{*}-U\right)+{\bar{\delta W}}_{2D}+{\bar{\delta W}}^{*}\right]dt=0,$$

### 3.1. First Approximation

Utilizing the VAM allows one to derive the leading terms in Equation (30) by disregarding smaller terms, such as

(31) $$\int_{t_{1}}^{t_{2}}\left[\delta \left(K_{2D}-\int_{\Omega }U_{0}d\Omega \right)+{\bar{\delta W}}_{2D}\right]dt=0,$$

where the first approximation of the strain energy can be obtained by eliminating smaller energy contributions due to $w_{i,\alpha }$, such as

(32) $$2U_{0}=\langle \begin{array}{c} {\left(\epsilon +\zeta y_{3}\kappa \right)}^{T}C_{e}\left(\epsilon +\zeta y_{3}\kappa \right)+2{\left(\epsilon +\zeta y_{3}\kappa \right)}^{T}\left(C_{es}w_{∥,3}+C_{et}w_{3,3}\right)\\ +w_{∥,3}^{T}C_{s}w_{∥,3}+2w_{∥,3}^{T}C_{st}w_{3,3}+w_{3,3}^{T}C_{t}w_{3,3} \end{array}\rangle .$$

Introducing a Lagrange multiplier $\lambda_{i}$ allows one to derive the associated Euler–Lagrange equation as

(33) $$\begin{array}{cc} \; & {\left[{\left(\epsilon +\zeta y_{3}\kappa \right)}^{T}C_{es}+w_{∥,3}^{T}C_{s}+w_{∥,3}^{T}C_{st}\right]}_{,3}=\lambda_{∥},\\ \; & {\left[{\left(\epsilon +\zeta y_{3}\kappa \right)}^{T}C_{et}+w_{∥,3}^{T}C_{st}+w_{3,3}C_{t}\right]}_{,3}=\lambda_{3}, \end{array}$$

where $\lambda_{\left|\right|}={\left[\lambda_{1}\;\lambda_{2}\right]}^{T}$.

The free boundary conditions at the top and bottom surfaces of the sandwich panel can be determined by

(34) $$\begin{array}{cc} \; & {\left[{\left(\epsilon +\zeta y_{3}\kappa \right)}^{T}C_{es}+w_{∥,3}^{T}C_{s}+w_{∥,3}^{T}C_{st}\right]}^{+/-}=0,\\ \; & {\left[{\left(\epsilon +\zeta y_{3}\kappa \right)}^{T}C_{et}+w_{∥,3}^{T}C_{st}+w_{3,3}C_{t}\right]}^{+/-}=0, \end{array}$$

where the superscript “+/−” indicates the quantity on the top and bottom surfaces of the panel, respectively.

Given these conditions, one can represent the solutions for $w_{\left|\right|}$ and $w_{3}$ as

(35) $$w_{∥}={\langle -\left(\epsilon +\zeta y_{3}\kappa \right){\bar{C}}_{es}{\left(C_{s}\right)}^{-1}\rangle }^{T},w_{3}=\langle -\left(\epsilon +\zeta y_{3}\kappa \right){\bar{C}}_{et}{\left({\bar{C}}_{t}\right)}^{-1}\rangle ,$$

where

(36) $${\bar{C}}_{es}=C_{es}-{\bar{C}}_{et}{\left(C_{st}\right)}^{T}{\left({\bar{C}}_{t}\right)}^{-1},{\bar{C}}_{et}=C_{et}-C_{es}{\left(C_{s}\right)}^{-1}C_{st},{\bar{C}}_{t}=C_{t}-{\left(C_{st}\right)}^{T}{\left(C_{s}\right)}^{-1}C_{st}.$$

The stain energy of the 2D-ERM can be determined by substituting Equation (35) in Equation (32):

(37) $$U_{2D}=\frac{1}{2}\langle {\left(\epsilon +\zeta y_{3}\kappa \right)}^{T}K\left(\epsilon +\zeta y_{3}\kappa \right)\rangle =\frac{1}{2}{\left\{\begin{array}{c} \epsilon \\ \kappa \end{array}\right\}}^{T}\left[\begin{array}{cc} A & B\\ B & D \end{array}\right]\left\{\begin{array}{c} \epsilon \\ \kappa \end{array}\right\},$$

with

(38) $$\begin{array}{c} A=\langle K\rangle ,B=\langle \zeta y_{3}K\rangle ,D=\langle \zeta y_{3}^{2}K\rangle ,\\ K=C_{e}-{\bar{C}}_{es}C_{s}^{-1}C_{es}^{T}-{\bar{C}}_{et}C_{et}^{T}/{\bar{C}}_{t}. \end{array}$$

### 3.2. Second Approximation

The first approximation aligns with classical plate theory, allowing for the calculation of in-plane stresses. However, to accurately consider out-of-plane stresses, the second approximation is necessary. This involves perturbing the warping function as

(39) $$w_{∥}={\bar{v}}_{∥},\;w_{3}={\bar{v}}_{3}+D_{⊥}w,$$

where $w={\left[\begin{array}{cc} \varepsilon & \kappa \end{array}\right]}^{T},D_{⊥}=\left[-\frac{D_{et}^{T}}{D_{t}}-x_{3}\frac{D_{et}^{T}}{D_{t}}\right]$.

Substituting Equation (39) into Equation (31) and then into Equation (32), the expression for the second approximate energy can be obtained as

(40) $$2\Pi_{1}=\langle {\bar{v}}_{∥,3}^{T}D_{s}{\bar{v}}_{∥,3}+D_{t}{\bar{v}}_{3,3}^{2}+2{\bar{v}}_{∥}^{T}C_{∥,3}w_{,\alpha }+2{\bar{v}}_{∥}^{T}D_{s}\partial_{t}D_{⊥}w_{,\alpha }-2{\bar{v}}_{∥}^{-T}p_{∥}\rangle -2{\bar{v}}_{∥}^{T}\tau_{∥}-2{\bar{v}}_{∥}^{T}\beta_{∥}.$$

The related Euler–Lagrange equation can be derived as

(41) $${\left(D_{s}{\bar{v}}_{∥,3}+D_{s}\partial_{t}D_{⊥}w_{,\alpha }\right)}_{,3}=C_{∥,3}\partial_{t}D_{⊥}w_{,\alpha }+g_{,3}+\lambda_{∥},$$

where $C_{∥}=-\partial_{e}^{T}\left[\begin{array}{cc} D_{∥} & x_{3}D_{∥} \end{array}\right],g_{,3}=-p_{∥}$.

Since ${\bar{v}}_{3}$ does not depend on ${\bar{v}}_{∥}$, ${\bar{v}}_{3}$ has a trivial solution. Consequently, the solution for ${\bar{v}}_{∥}$ can be determined as

(42) $${\bar{v}}_{∥}=\left({\bar{C}}_{∥}+L_{\alpha }\right)w_{,\alpha }+\bar{g},$$

where

(43) $$\begin{array}{c} {\bar{C}}_{∥,3}=D_{s}^{-1}C_{∥},\;\langle {\bar{C}}_{∥}\rangle =0,\;{\bar{g}}_{3}=D_{s}^{-1}\bar{g},\;\langle \bar{g}\rangle =0,\\ L_{\alpha }w_{,\alpha }={\bar{C}}_{∥}/h,\;{\bar{C}}_{∥}=C_{∥}+\frac{x_{3}}{h}D_{\alpha }^{\mp }-\frac{1}{2}D_{∥}^{\pm }-D_{s}e_{\alpha }D_{⊥},\bar{g}=g+\frac{x_{3}}{h}g^{\mp }-\frac{1}{2}g^{\pm }, \end{array}$$

and ${\left(\cdot \right)}^{\mp }={\left(\cdot \right)}^{-}-{\left(\cdot \right)}^{+},{\left(\cdot \right)}^{\pm }={\left(\cdot \right)}^{+}+{\left(\cdot \right)}^{-}$.

The second-order approximate energy can be formulated in the form of the Reissner-Mindlin model as

(44) $$2\Pi_{1}=w^{T}Aw+w_{,\alpha }^{T}B_{\alpha \beta }w_{,\beta }-2w^{T}F,$$

where $B_{\alpha \beta }$ is additional shear stiffness and F is a load-related item:

(45) $$\begin{array}{c} A=\left[\begin{array}{cc} \langle D_{∥}\rangle & \langle x_{3}D_{∥}\rangle \\ \langle x_{3}D_{∥}\rangle & \langle x_{3}^{2}D_{∥}\rangle \end{array}\right],B_{\alpha \beta }=\langle D_{S_{\alpha \beta }}D_{⊥}^{T}D_{⊥}-{\bar{C}}_{\alpha }^{T}D_{s}^{-1}{\bar{C}}_{\beta }\rangle +L_{\alpha }^{T}\left(C_{\beta ,3}\right),\\ F=\langle D_{⊥}^{T}p_{3}\rangle -\langle {\bar{C}}_{∥}^{T}D_{s}^{-1}{\bar{g}}_{,a}\rangle -L_{\alpha }{\left(\langle \bar{p}\rangle +\langle p_{∥}\rangle \right)}_{,\alpha }. \end{array}$$

The constitutive relationship for the 2D-ERM can be derived as

(46) $$\left\{\begin{array}{c} N_{11}\\ N_{22}\\ N_{12}\\ M_{11}\\ M_{22}\\ M_{12}\\ Q_{1}\\ Q_{2} \end{array}\right\}=\left[\begin{array}{cccccccc} A_{11} & A_{12} & A_{16} & B_{11} & B_{12} & B_{16} & Y_{11} & Y_{12}\\ A_{12} & A_{22} & A_{26} & B_{12} & B_{22} & B_{26} & Y_{21} & Y_{22}\\ A_{16} & A_{26} & A_{66} & B_{16} & B_{26} & B_{66} & Y_{31} & Y_{32}\\ B_{11} & B_{12} & B_{16} & D_{11} & D_{12} & D_{16} & Y_{41} & Y_{42}\\ B_{12} & B_{22} & B_{26} & D_{12} & D_{22} & D_{26} & Y_{51} & Y_{52}\\ B_{16} & B_{26} & B_{66} & D_{16} & D_{26} & D_{66} & Y_{61} & Y_{62}\\ Y_{11} & Y_{21} & Y_{31} & Y_{41} & Y_{51} & Y_{61} & G_{11} & G_{12}\\ Y_{12} & Y_{22} & Y_{32} & Y_{42} & Y_{22} & Y_{62} & G_{12} & G_{22} \end{array}\right]\left\{\begin{array}{c} \epsilon_{11}\\ \epsilon_{22}\\ 2\epsilon_{12}\\ \kappa_{11}\\ \kappa_{22}\\ 2\kappa_{12}\\ 2\gamma_{13}\\ 2\gamma_{23} \end{array}\right\}$$

where $N_{\alpha \beta }$, $M_{\alpha \beta }$, and $Q_{\alpha }$ are the in-plane force, moment, and transverse shear stress resultants, respectively.

## 4. Free- and Forced-Vibration Analysis of the Damped 2D-ERM

The free-vibration equation of the 2D-ERM is

(47) $$D_{1}\frac{\partial^{2}\psi_{1}}{\partial x_{1}^{2}}+\frac{\partial^{2}\psi_{1}}{\partial x_{2}^{2}}+D_{3}\frac{\partial^{2}\psi_{2}}{\partial x_{1}\partial x_{2}}+C_{1}\left(\frac{\partial {\bar{u}}_{3}}{\partial x_{1}}-\psi_{1}\right)+\gamma^{4}\psi_{1}=0$$

(48) $$D_{2}\frac{\partial^{2}\psi_{2}}{\partial x_{2}^{2}}+\frac{\partial^{2}\psi_{y}}{\partial x^{2}}+D_{3}\frac{\partial^{2}\psi_{1}}{\partial x_{1}\partial x_{2}}+C_{2}\left(\frac{\partial {\bar{u}}_{3}}{\partial x_{2}}-\psi_{2}\right)+\gamma^{4}\psi_{2}=0$$

(49) $$C_{1}\left(\frac{\partial^{2}{\bar{u}}_{3}}{\partial x_{1}^{2}}-\frac{\partial \psi_{1}}{\partial x_{1}}\right)+C_{2}\left(\frac{\partial^{2}{\bar{u}}_{3}}{\partial x_{2}^{2}}-\frac{\partial \psi_{2}}{\partial x_{2}}\right)+\beta^{4}{\bar{u}}_{3}=0$$

where

(50) $$\begin{array}{c} D_{1}=\frac{D_{11}}{D_{66}},\;D_{2}=\frac{D_{22}}{D_{66}},\;D_{3}=\frac{D_{12}+D_{66}}{D_{66}},\;C_{1}=\frac{C_{55}}{D_{66}},\;C_{2}=\frac{C_{44}}{D_{66}},\\ \gamma^{4}=\frac{\rho^{*}J\omega^{2}}{D_{66}},\;\beta^{4}=\frac{\rho^{*}h\omega^{2}}{D_{66}} \end{array}$$

Differentiating Equation (49) with respect to $x_{1}$ and $x_{2}$, and substituting the results into Equations (47) and (48), respectively, to eliminate $\psi_{1}$ and $\psi_{2}$, one obtains

(51) $$\left[1-\frac{1}{C_{1}}\left(D_{1}\frac{\partial^{2}}{\partial x_{1}^{2}}-D_{3}\frac{C_{1}}{C_{2}}\frac{\partial^{2}}{\partial x_{1}^{2}}+\frac{\partial^{2}}{\partial x_{2}^{2}}+\gamma^{4}\right)\right]\psi_{1}=\left[1+\frac{D_{3}}{C_{1}}\left(\frac{\partial^{2}}{\partial x_{2}^{2}}+\frac{C_{1}}{C_{2}}\frac{\partial^{2}}{\partial x_{1}^{2}}+\frac{\beta^{4}}{C_{2}}\right)\right]\frac{\partial {\bar{u}}_{3}}{\partial x_{1}}$$

(52) $$\left[1-\frac{1}{C_{2}}\left(D_{2}\frac{\partial^{2}}{\partial x_{2}^{2}}-D_{3}\frac{C_{2}}{C_{1}}\frac{\partial^{2}}{\partial x_{2}^{2}}+\frac{\partial^{2}}{\partial x_{1}^{2}}+\gamma^{4}\right)\right]\psi_{2}=\left[1+\frac{D_{3}}{C_{2}}\left(\frac{\partial^{2}}{\partial x_{1}^{2}}+\frac{C_{2}}{C_{1}}\frac{\partial^{2}}{\partial x_{2}^{2}}+\frac{\beta^{4}}{C_{1}}\right)\right]\frac{\partial {\bar{u}}_{3}}{\partial x_{2}}$$

Equation (49) can be rewritten as

(53) $$\left(C_{1}\frac{\partial^{2}}{\partial x_{1}^{2}}+C_{2}\frac{\partial^{2}}{\partial x_{2}^{2}}+\beta^{4}\right){\bar{u}}_{3}=C_{1}\frac{\partial \psi_{1}}{\partial x_{1}}+C_{2}\frac{\partial \psi_{2}}{\partial x_{2}}$$

The damped forced-vibration equilibrium equation for the 2D-ERM is

(54) $$-\frac{\partial M_{11}}{\partial x_{1}}-\frac{\partial M_{12}}{\partial x_{2}}+Q_{1}-\rho^{*}J\frac{\partial^{2}\psi_{1}}{\partial t^{2}}=0,$$

(55) $$-\frac{\partial M_{12}}{\partial x_{1}}-\frac{\partial M_{22}}{\partial x_{2}}+Q_{2}-\rho^{*}J\frac{\partial^{2}\psi_{2}}{\partial t^{2}}=0,$$

(56) $$\frac{\partial Q_{1}}{\partial x_{1}}+\frac{\partial Q_{2}}{\partial x_{2}}+p=\rho^{*}h\frac{\partial^{2}{\bar{u}}_{3}}{\partial t^{2}}+c\frac{\partial {\bar{u}}_{3}}{\partial t}.$$

where the load *p* is only a function of time *t*, and *c* is the damping coefficient.

By substituting $M_{11},\;M_{22},\;M_{12},\;Q_{1}$, and $Q_{2}$ into Equations (54)–(56), one obtains the differential equation of damped forced vibration as

(57) $$D_{11}\frac{\partial^{2}\psi_{1}}{\partial x_{1}^{2}}+D_{66}\frac{\partial^{2}\psi_{1}}{\partial x_{2}^{2}}+\left(D_{12}+D_{66}\right)\frac{\partial^{2}\psi_{2}}{\partial x_{1}\partial x_{2}}+C_{55}\left(\frac{\partial {\bar{u}}_{3}}{\partial x_{1}}-\psi_{1}\right)-\rho^{*}J\frac{\partial^{2}\psi_{1}}{\partial t^{2}}=0,$$

(58) $$D_{22}\frac{\partial^{2}\psi_{2}}{\partial x_{2}^{2}}+D_{66}\frac{\partial^{2}\psi_{2}}{\partial x_{1}^{2}}+\left(D_{12}+D_{66}\right)\frac{\partial^{2}\psi_{1}}{\partial x_{1}\partial x_{2}}+C_{44}\left(\frac{\partial {\bar{u}}_{3}}{\partial x_{2}}-\psi_{2}\right)-\rho^{*}J\frac{\partial^{2}\psi_{2}}{\partial t^{2}}=0,$$

(59) $$C_{55}\left(\frac{\partial^{2}{\bar{u}}_{3}}{\partial x_{1}^{2}}-\frac{\partial \psi_{1}}{\partial x_{1}}\right)+C_{44}\left(\frac{\partial^{2}{\bar{u}}_{3}}{\partial x_{2}^{2}}-\frac{\partial \psi_{2}}{\partial x_{2}}\right)+p=\rho^{*}h\frac{\partial^{2}{\bar{u}}_{3}}{\partial t^{2}}+c\frac{\partial {\bar{u}}_{3}}{\partial t}.$$

where

(60) $$C_{44}=\kappa G_{11}h,\;C_{55}=\kappa G_{22}h.$$

Based on the vibration modes and natural frequencies obtained from free-vibration analysis, the locations of receiver and excitation points under dynamic loading, such as harmonic loading, are determined based on the method outlined in Ref. [32]. Specifically, the obtained effective properties of the damped BHC-CSP are incorporated into the equivalent model through the ’general shell’ feature in the S4R element, serving as an enhancement to the commercial program. Moreover, the ’frequency’ procedure in ABAQUS software (version 6.14) is utilized to determine the natural frequencies and vibration modes. Additionally, the modal/steady-state dynamics solver in ABAQUS software is employed for time- and frequency-domain forced-vibration analysis, respectively.

## 5. Free- and Forced-Vibration Analysis of Damped BHC-CSP

**Figure 5:**
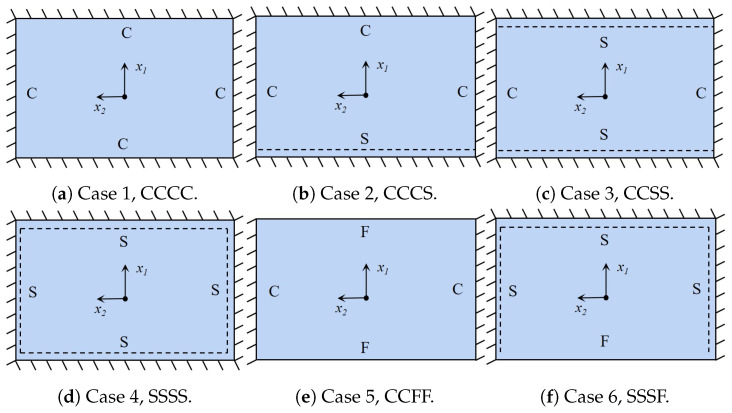
Schematic diagram of different boundary conditions for free-vibration analysis.

The dynamic performance of the damped BHC-CSP, particularly regarding free- and forced-vibration characteristics, plays a pivotal role in ensuring structural reliability and durability. The addition of damping layers to the sandwich panel introduces significant complexity, rendering analytical solutions and experimental data from the existing literature unavailable. Therefore, this section relies on numerical simulations as a valuable substitute to investigate the free and forced vibration of the damped BHC-CSP, providing insights that may inform future experimental endeavors and potentially result in substantial time and resource savings. Six typical boundary conditions (CCCC, CCCS, CCSS, SSSS, CCFF, and SSSF), as outlined in Figure 5, are used to investigate its free-vibration characteristics, with the letters C, S, and F denoting the clamped, simply supported, and free sides, respectively.

### 5.1. Model Information

**Figure 6:**
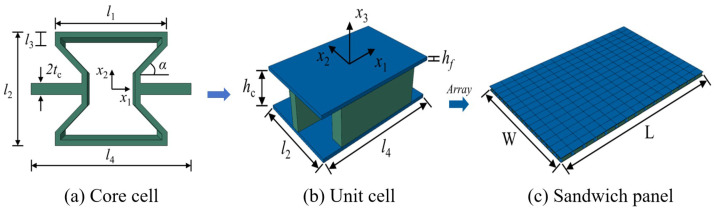
Composition of BHC-CSP.

Figure 6a illustrates the core cell of the BHC-CSP, featuring the following structural specifications: $t_{c}$ = 0.8 mm, $l_{1}$ = 16.8 mm, $l_{2}$ = 16 mm, $l_{3}$ = 2.4 mm, and $l_{4}$ = 24 mm. In addition, the core height is $h_{c}$ = 6 mm while the face panel thickness is $h_{f}$ = 0.6 mm. The BHC-CSP consists of 15 unit cells in both $x_{1}$ and $x_{2}$ directions, with dimensions of *L* = 360 mm in length and *W* = 240 mm in width. The top and bottom facesheets are composed of CFRP material with a layup configuration of [45/−45/0/90]*_s_* and a fiber volume fraction of 60%. The core layer is constructed from aluminum metal. Detailed properties of each material can be referenced in Table 1.

**Figure 7:**
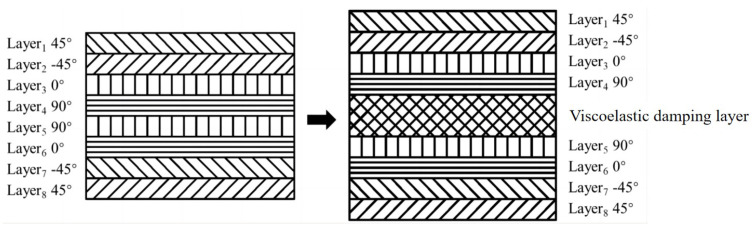
Laminated structure of viscous layers and composite materials.

The viscoelastic materials are Polyimide (PMI) foam with a higher modulus and ternary ethylene propylene rubber foam (T-SW308) with a lower modulus, with their material properties detailed in Table 1. It can be anticipated that the developed method is suitable for other damping materials with differing moduli. A previous study indicates that incorporating viscoelastic damping layers on the facesheet and core layer individually had similar effects on the overall structural stiffness. However, positioning the damping layer in the facesheet led to superior enhancement of the damping performance of the sandwich panel. This superiority can be attributed to the higher strains experienced by the top and bottom facesheets of the sandwich panel during torsional or bending deformations. Therefore, the viscoelastic damping layer is integrated into the facesheet, as illustrated in Figure 7. It is mentioned that the 3D-FEM utilizes 340,653 C3D10 elements and 600,301 nodes, employing a structured meshing approach with a mesh size set to 0.2 mm after a mesh convergence study.

### 5.2. Free-Vibration Analysis of BHC-CSP with Viscoelastic Damping Layer

**Figure 8:**
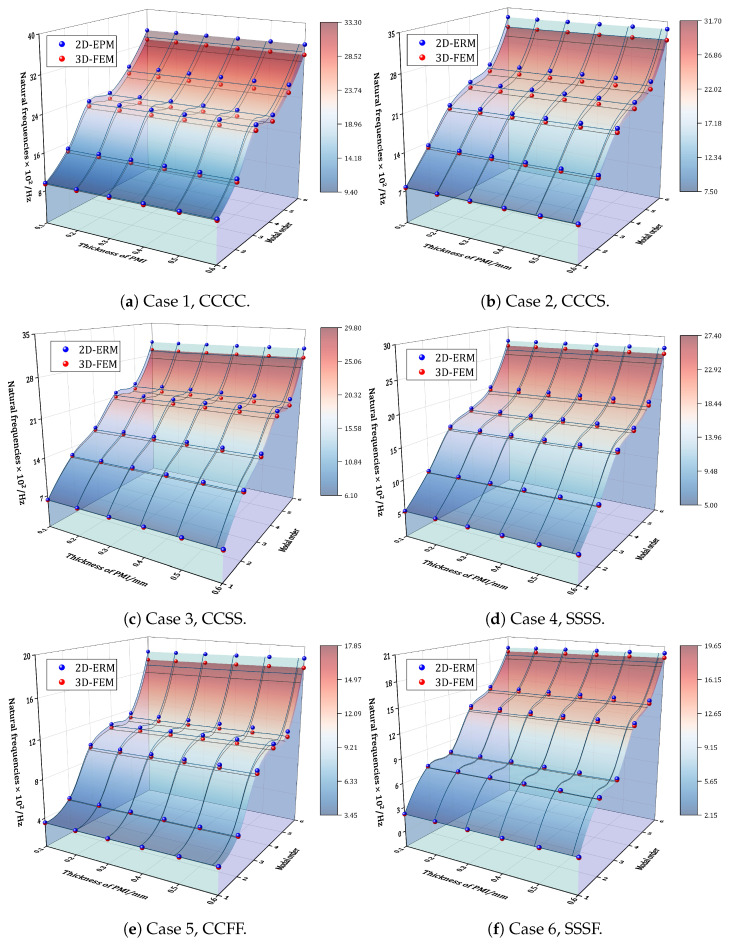
Effect of varying thicknesses of PMI damping layers on the natural frequency of BHC-CSP.

This section investigates the impact of PMI damping layers on the natural frequencies of composite sandwich panels. Figure 8 compares the first six natural frequencies of the BHC-CSP with varying thicknesses of the PMI damping layers under six different cases, as predicted by the 2D-ERM and 3D-FEM. Specifically, the red dots correspond to the 3D-FEM data, while the blue dots correspond to the 2D-ERM data. The results indicate that the VAM-based 2D-ERM accurately captures the impact of the viscoelastic damping layer on the natural frequencies. The natural frequencies exhibit a gradual increase with variations in the PMI foam thickness from 0.1 to 0.6 mm. Notably, the increase in higher-order natural frequencies is proportionally comparable to that of the lower-order natural frequencies. This behavior stems from PMI foam with a higher modulus; the addition of a PMI layer effectively increases the panel thickness, thereby augmenting the overall structural stiffness. Despite the rise in the sandwich panel’s total mass due to the inclusion of PMI foam, this increase in mass fails to counterbalance the amplified natural frequencies resulting from enhanced stiffness. Particularly when the PMI damping layer is thin, the effect of stiffness enhancement on natural frequencies becomes more discernible.

In addition, the relatively uniform distribution of PMI foam within the facesheet of the sandwich panel results in a uniform effect on the stiffness and mass of the entire panel, thereby exerting comparable influences on both higher- and lower-order frequencies. To sum up, integrating a constrained viscoelastic damping layer allows for a precise adjustment of the stiffness and mass distribution within the sandwich panel, laying the groundwork for the tailored design of panels with distinct vibration characteristics.

### 5.3. Frequency-Domain Analysis of BHC-CSP with Viscoelastic Damping Layer

Forced-vibration analysis assesses the panel’s response to external dynamic loads, a critical step in evaluating and optimizing the panel’s performance in its operational environment. This section investigates the impact of various viscoelastic damping layers on the resonant response of the BHC-CSP. The modal dynamic analysis involves single-point excitation and output to capture additional resonance responses for the first six frequencies under SSSS BCs.

**Figure 9:**
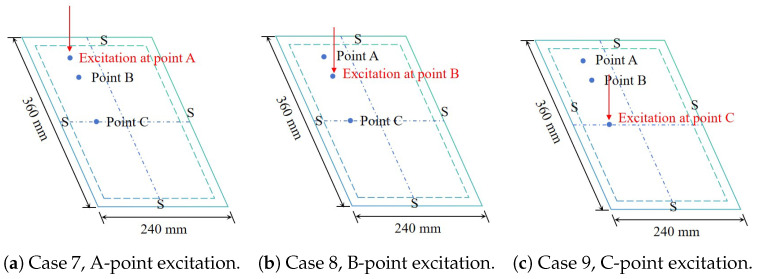
Schematic diagram of excitation location on the sandwich panel under SSSS BCs.

The excitation force, set as a harmonic load with a 10 kN amplitude, sweeps across frequencies ranging from 0 to 3000 Hz, with a damping ratio of 0.02 for each mode. To explore the impact of excitation location on the structural frequency-domain response, three receiving points—A, B, and C—at coordinates (45, 60), (90, 60), and (180, 60), respectively, are selected for investigation, as shown in Figure 9. Through the steady-state modal dynamic analysis step in the ABAQUS software, a frequency-domain response analysis is conducted on the sandwich panel with or without these viscoelastic layers.

**Figure 10:**
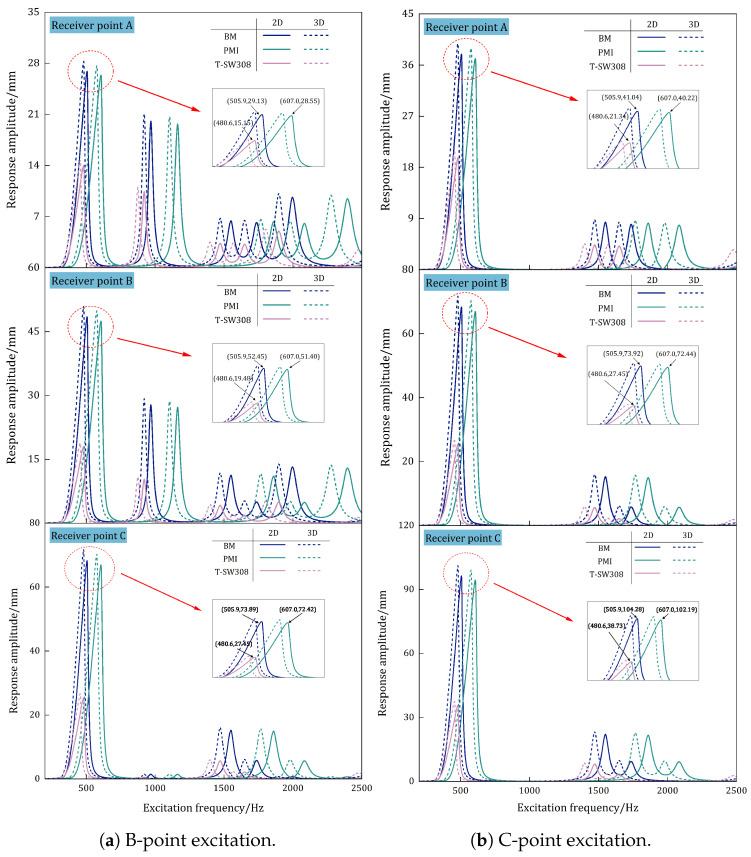
Effect of viscous damping layers on frequency-domain response of damped BHC-CSP.

Figure 10a,b compare the frequency-domain response predicted by the 3D-FEM and 2D-ERM under Cases 8 and 9, where points B and C are excited. The frequency-domain response curves are obtained at three distinct receiving points for six models (BM, BM-2D, PMI, PMI-2D, T-SW308, and T-SW308-2D). In this context, BM, PMI, and T-SW308 represent the BHC-CSP without the viscoelastic damping layer and with the PMI and T-SW308 viscoelastic layers, respectively. On the other hand, BM-2D, PMI-2D, and T-SW308-2D represent the 2D equivalent models of the corresponding sandwich panels.

It is evident that the equivalent model is applicable for studying the frequency-domain response of the damped BHC-CSP. Specifically, the PMI-2D and T-SW308-2D models can effectively predict the resonant response of the corresponding viscoelastic-damped sandwich panels while meeting engineering accuracy requirements. An examination of the first-order resonance amplitudes reveals that the addition of the PMI foam viscoelastic layer has an insignificant impact on the panel’s resonance amplitude, while incorporating the T-SW308 foam viscoelastic layer effectively reduces the panel’s resonance amplitude. Adding the PMI foam viscoelastic layer results in an increase of 19.98% in the fundamental frequency, whereas adding the T-SW308 foam viscoelastic layer leads to a 5.00% decrease in the fundamental frequency. This is attributed to the soft texture of T-SW308 foam, which to some extent reduces the panel’s stiffness, consequently lowering the natural frequency.

In summary, a comprehensive comparison shows that the PMI damping layer can effectively increase the panel’s stiffness with minimal impact on the damping performance, while the T-SW308 damping layer can significantly enhance the damping performance without significantly altering the stiffness of the sandwich panel. This indicates that T-SW308 foam is an excellent damping layer material with a superior damping performance.

### 5.4. Time-Domain Analysis of BHC-CSP with Viscoelastic Damping Layer

**Figure 11:**
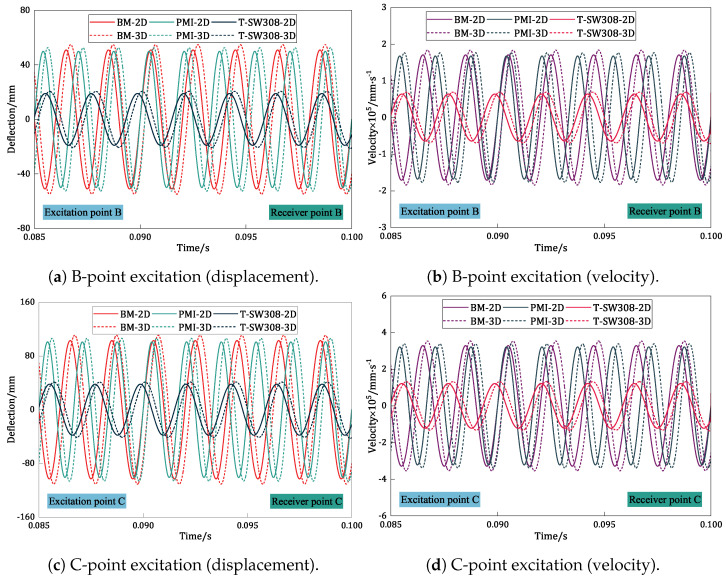
Effect of viscous damping layers on the time-domain response of damped BHC-CSP.

The time-domain responses of the sandwich panel with the PMI damping layer, T-SW308 damping layer, and without a viscoelastic damping layer are examined by applying excitations of $F=-10\sin2\pi f_{i}t$ at points B and C, as shown in Figure 11. The results indicate that the inclusion of the T-SW308 damping layer leads to a significant reduction in the displacement and velocity amplitudes of the sandwich panel. This reduction suggests a notable decrease in the overall system energy, indicating that the viscoelastic damping layer effectively dampens vibration amplitudes, reducing the likelihood of resonant amplification and structural damage.

Moreover, following the addition of the viscoelastic damping layer, the displacement–time and velocity–time histories exhibit a tendency to stabilize more rapidly. This illustrates that the viscoelastic damping layer aids in dissipating vibrational energy more efficiently, enabling the system to achieve an equilibrium state in a shorter duration. However, excessive damping can result in the over-dissipation of vibrational energy, potentially slowing down the free decay process of vibrations. Consequently, in engineering practice, it is critical to comprehensively account for other factors when enhancing the damping performance. This approach ensures a balanced consideration of both stability and sensitivity in the panel, ultimately facilitating an optimal system performance in vibrational settings.

### 5.5. Comparison of Dynamic Analysis Efficiency

The numerical simulations were carried out on the Dell Precision 3660 workstation. Table 2 delineates a thorough comparison of the computational efficiency between the two models utilized for dynamic analysis. It illustrates that the time needed for free vibration and forced vibration in the frequency and time domain using the 2D-ERM is notably lower in comparison to the 3D-FEM, showcasing reductions of 1.9%, 2.6%, and 1.6%, respectively. These results emphasize the significant improvement in dynamic analysis efficiency through the VAM-based 2D-ERM, presenting a more efficient tool for dynamic performance evaluation in engineering fields.

The comparison highlights the superior efficiency and accuracy of the 2D-ERM in quantitatively assessing the free- and forced-vibration behavior of the BHC-CSP with the viscoelastic damping layer. However, in three-dimensional models with intricate geometric and physical characteristics, the limitations of the 2D-ERM become apparent, as it fails to capture the detailed geometric intricacies of complex structures as comprehensively as the 3D-FEM. Consequently, when selecting a model for dynamic analysis, it is essential to strike a balance between model accuracy and computational efficiency.

## 6. Parameters-Influenced Analysis

This section examines the influence of four crucial geometric parameters on the natural frequency, equivalent density, and displacement–time history of the damped BHC-CSP utilizing the 2D-ERM. The alterations in geometric parameters are detailed in Table 3 for reference.

### 6.1. Height Ratio ($h_{c}/h_{f}$)

**Figure 12:**
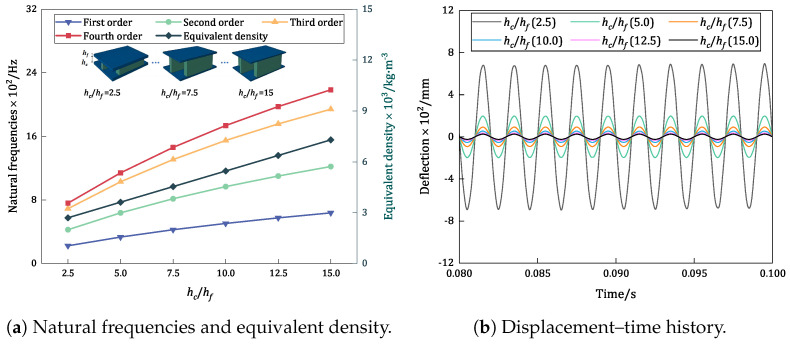
Influence of height ratio of core to facesheet on the vibration characteristics of damped BHC-CSP.

Figure 12 compares the trends in the variation in the first four natural frequencies, equivalent densities, and displacement–time history of the damped BHC-CSP with different height ratios. The examination of natural frequencies illustrates a nonlinear rise in the first four natural frequencies as the core layer height increases, with the growth rate gradually declining. This phenomenon stems from a more pronounced escalation in equivalent stiffness relative to equivalent density, resulting in a significant upsurge in the natural frequency. The declining growth rate indicates reduced benefits of further heightening the core layer beyond a certain threshold.

Figure 12b indicates that with an increase in the core layer height, the panel exhibits decreased sensitivity to external excitation, leading to a gradual decline in the resonance amplitude. This decline is attributed to the marked augmentation in overall structural stiffness with an increased core layer height, resulting in a more dispersed vibration energy and amplified damping effects, consequently reducing the resonance amplitude. These findings underscore the effectiveness of adjusting the core layer height in enhancing the resonance response of sandwich panels.

### 6.2. Wall Thickness-to-Height Ratio ($t_{c}/h_{c}$)

**Figure 13:**
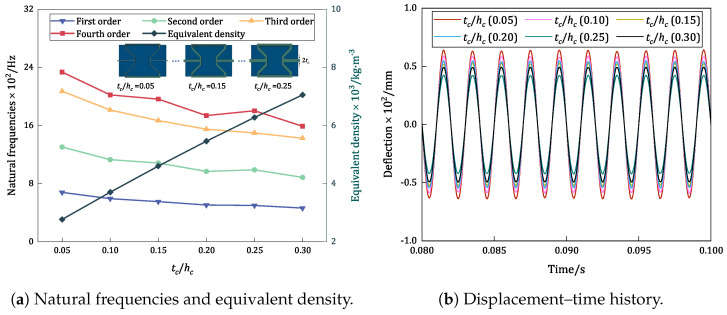
Influence of wall thickness-to-height ratio on the vibration characteristics of damped BHC-CSP.

Figure 13 compares the trends in the variations in the first four natural frequencies, equivalent densities, and displacement–time history curves of the damped BHC-CSP with different wall thickness-to-height ratios. An examination of the natural frequencies indicates that as the wall thickness increases, there is a decreasing trend in natural frequency, with a slight mutation observed at the ratio of 0.25. This decrease is attributed to a sudden increase in equivalent stiffness at this ratio, which surpasses the increase in equivalent density, resulting in an increase in natural frequency (the equivalent density, equivalent tensile stiffness, and equivalent bending stiffness increase by 15%, 105%, and 66%, respectively). Figure 13b illustrates that the resonance amplitude gradually decreases with an increase in the cell wall thickness. This reduction can be attributed to the corresponding increase in the structure’s equivalent stiffness, particularly the shear stiffness and torsional stiffness, both crucial for resisting shear and torsional deformations, which commonly constitute significant components of the vibration modes.

### 6.3. Re-Entrant Angle (θ)

**Figure 14:**
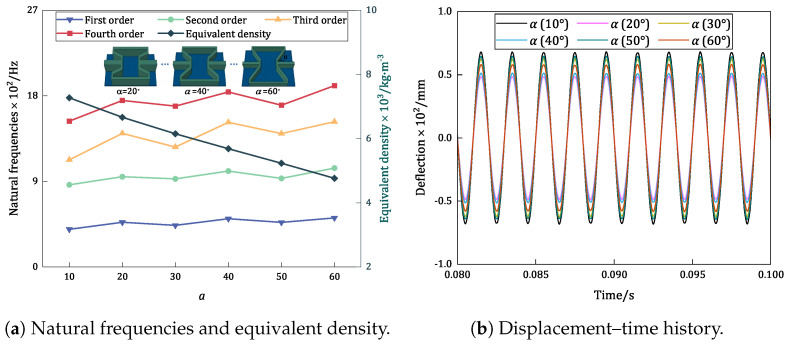
Influence of re-entrant angle on the vibration characteristics of damped BHC-CSP.

Figure 14 compares the trends in the variations in the first four natural frequencies, equivalent densities, and displacement–time history curves of the damped BHC-CSP with different re-entrant angles. An examination of the natural frequencies reveals a gradual decrease in equivalent density as the re-entrant angle increases, while the changes in natural frequency align with the variations in directional equivalent stiffness. An investigation of the resonance response in Figure 14b indicates that the resonance amplitude of the panel is more sensitive to changes in directional equivalent stiffness. A detailed comparison of resonance response and natural frequencies indicates that the panel demonstrates higher natural frequencies and relatively smaller resonance amplitudes at $\alpha =40^{\circ }$, mitigating the risk of external excitation-induced resonance failure.

### 6.4. Aspect Ratio ($l_{4}/l_{2}$)

**Figure 15:**
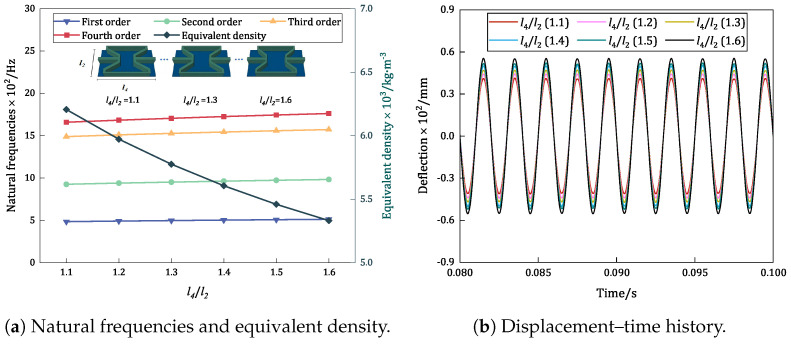
Influence of aspect ratio on the vibration characteristics of damped BHC-CSP.

Figure 15 compares the trends in the variations in the first four natural frequencies, equivalent densities, and displacement–time history curves of the damped BHC-CSP with different aspect ratios. An examination of the natural frequencies unveils that the first four natural frequencies rise as the aspect ratio increases. This increase is attributed to the smaller decrease in structural equivalent stiffness (8.88%) compared to the decrease in equivalent density (14.09%). An examination of the resonance response in Figure 15b reveals that the resonance amplitude of the panel escalates with the aspect ratio increment. This phenomenon can be explained by the panel becoming more flexible as the overall stiffness decreases, allowing for increased energy absorption during vibration and consequently leading to a larger dynamic response during resonance. Notably, as the $l_{4}/l_{2}$ ratio shifts from 1.1 to 1.6, the fundamental frequency of the panel increases by 5.72%, while the resonance amplitude experiences a 4.24% increase. These findings underscore the importance of considering the aspect ratio’s influence on the dynamic performance of the damped BHC-CSP.

### 6.5. Summary

To visually demonstrate the influence of geometric parameters on the dynamic performance of the damped BHC-CSP, Table 4 lists the unit changes in the equivalent density (Δρ), fundamental frequency (Δω), resonance amplitude ($\Delta R_{A}$), and equivalent tensile and bending stiffness ($\Delta A_{22}$ and $\Delta D_{22}$, respectively) with the variation in different parameters. It is evident that the core layer height exerts the most significant influence on the equivalent bending stiffness, fundamental frequency, and resonance amplitude. This observation suggests that adjusting the core layer height can effectively enhance the panel’s dynamic response to external excitations. Moreover, variations in the re-entrant angle, which impact the volume of the core layer per unit length, play a crucial role in adjusting the panel’s tensile performance. By adjusting the equivalent stiffness, the panel’s response efficiency to external excitations can be improved, leading to reduced vibration amplitudes and the effective mitigation of structural fatigue and resonance damage risks. Therefore, optimizing the equivalent stiffness and overall dynamic response can be achieved through adjustments to the re-entrant angle of the bowtie-shaped honeycomb.

## 7. Comparison of Vibration Characteristics between Different Honeycomb Sandwich Panels

Distinct geometric shapes of various honeycomb structures result in unique energy distribution, load transfer paths, and deformation modes. Analyzing the differences in the vibration characteristics of diverse honeycomb sandwich panels aids in enhancing the structural design rationale. This, in turn, facilitates the development of honeycomb structures with superior adaptability and mechanical properties.

**Figure 16:**
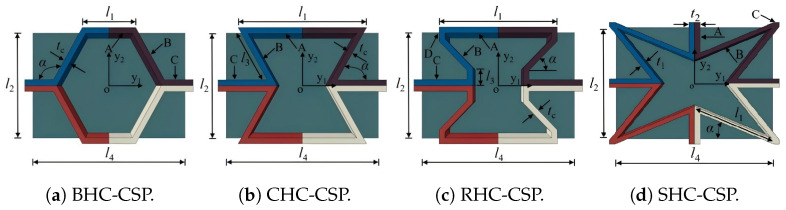
Different honeycomb sandwich panels.

In this section, apart from the BHC-CSP, three typical honeycomb sandwich panels are investigated, including the conventional hexagonal core sandwich panel (CHC-CSP), re-entrant hexagonal core sandwich panel (RHC-CSP), and star-shaped honeycomb core sandwich panel (SHC-CSP), as illustrated in Figure 16. The geometric parameters are as follows: CHC-CSP, $l_{1}=7.6\;\mathrm{mm},\;\alpha =120^{\circ }$; RHC-CSP, $l_{1}=18.3\;\mathrm{mm},\;\alpha =60^{\circ }$, $l_{3}=8.32\;\mathrm{mm}$; and SHC-CSP, $\alpha =30^{\circ },\;l_{1}=12.6\;\mathrm{mm}$. The geometric parameters for the bowtie honeycomb remain consistent with those outlined in Section 5.1. The layup mode of CFRP is [±45/0/90]*_s_*, with a fiber volume fraction of 60%, while the core layer material remains unchanged. The core layer weights for the hexagonal honeycomb, re-entrant honeycomb, bowtie-shaped honeycomb, and star-shaped honeycomb were 1.29 g, 1.27 g, 0.84 g, and 2.03 g, respectively. The core volumes were 477.25 mm^3^, 469.93 mm^3^, 310.57 mm^3^, and 750.54 mm^3^, respectively.

### 7.1. Comparison of Free-Vibration Characteristics

**Figure 17:**
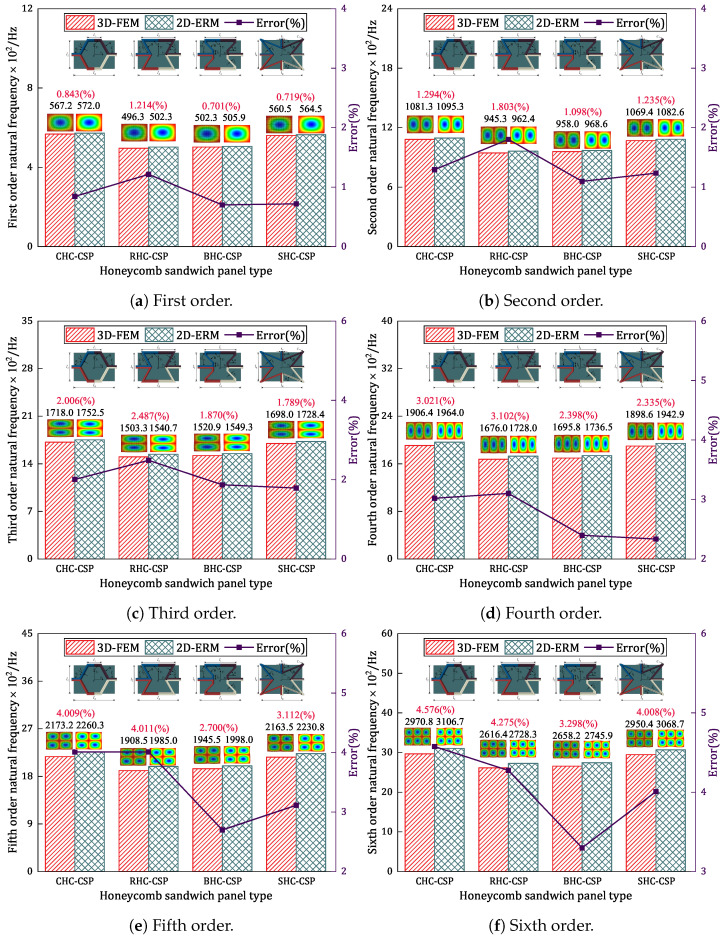
Comparison of natural frequencies for different honeycomb sandwich panels.

Figure 17a–f compare the first six natural frequencies of four damped honeycomb sandwich panels under SSSS BCs predicted by the 3D-FEM and 2D-ERM. It is observed that the VAM-based equivalent model can accurately predict the vibration modes of the respective damped honeycomb sandwich panel. While the relative errors of natural frequencies predicted by the equivalent model increase with the modal order, the relative errors fall between 0.701% and 4.576%. This finding confirms the precision of utilizing the VAM for analyzing the free-vibration characteristics of damped honeycomb sandwich panels with distinct honeycomb cores. Comparing the natural frequencies of the different damped honeycomb sandwich panels reveals that the fundamental frequency of the CHC-CSP, SHC-CSP, and BHC-CSP increases by 14.286%, 12.936%, and 1.209%, respectively, relative to the RHC-CSP. This suggests that the RHC-CSP is more susceptible to external excitation and resonance. In contrast, the CHC-CSP and SHC-CSP exhibit lower sensitivity to external excitation and demonstrate a more stable performance.

### 7.2. Comparison of Forced-Vibration Characteristics

**Figure 18:**
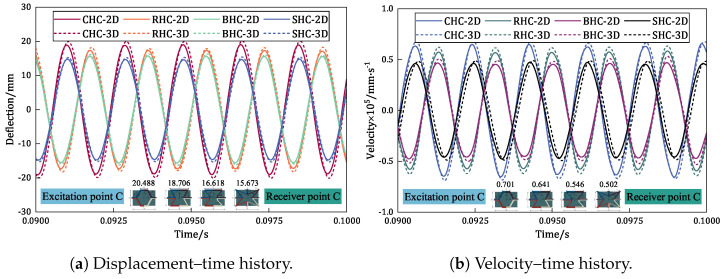
Comparison of time-domain response for different honeycomb sandwich panels.

The displacement–time and velocity–time history curves at point C of different damped honeycomb sandwich panels (the CHC-CSP, RHC-CSP, and BHC-CSP) were compared in Figure 18. Upon analyzing the displacement–time history curves, it is evident that the steady-state maximum displacement amplitudes of the CHC-CSP, RHC-CSP, and BHC-CSP in relation to the SHC-CSP increased by 30.719%, 19.350%, and 6.025%, respectively. This suggests that the CHC-CSP exhibits the highest increase in displacement amplitude, followed by the RHC-CSP and then the BHC-CSP.

Similarly, the steady-state maximum velocity amplitudes of the CHC-CSP, RHC-CSP, and BHC-CSP relative to the SHC-CSP increased by 39.627%, 27.733%, and 8.683%, respectively. This indicates that the CHC-CSP also shows the largest increase in velocity amplitude, followed by the RHC-CSP and then the BHC-CSP. These findings further elucidate the differences in vibration characteristics among the different damped honeycomb sandwich panels, with the CHC-CSP showcasing the highest displacement and velocity amplitudes, followed by the RHC-CSP and BHC-CSP.

It is important to highlight that the displacement–time history predicted by the VAM-based 2D-ERM aligns closely with those derived from the corresponding 3D-FEM, meeting the required engineering precision. The CHC-CSP is relatively insensitive to external excitation. However, it exhibits the largest displacement and velocity amplitudes during resonance, making the panel highly susceptible to damage induced by resonance. On the other hand, the BHC-CSP not only displays low sensitivity to external excitation but also demonstrates smaller displacement and velocity amplitudes during resonance. This suggests that the bowtie-shaped honeycomb design can effectively mitigate resonance effects and possesses a favorable anti-vibration performance.

In summary, while the CHC-CSP shows resilience to external excitations, its susceptibility to resonance-induced damage is a concern. In contrast, the BHC-CSP not only resists external excitations but also minimizes the impact of resonance, highlighting its superior anti-vibration capabilities.

## 8. Conclusions

This work aims to utilize the VAM to decompose the BHC-CSP with a viscoelastic damping layer into a unit-cell-based constitutive model and two-dimensional equivalent Reissner-Mindlin model (2D-ERM), enabling the analysis of the free- and forced-vibration characteristics of the damped BHC-CSP. The efficiency and accuracy of the 2D-ERM were assessed by comparing the results from the 3D-FEM. The key conclusions drawn from the study are as follows:

(1) Reinforcing the boundary constraints diminishes the effective vibration region of the panel, necessitating higher frequencies to attain equivalent amplitudes. The increase in the panel’s mass resulting from the inclusion of a PMI damping layer is less pronounced compared to the augmentation in stiffness. Given the relatively uniform distribution of the PMI damping layer within the panel, its impact on both high-order and low-order vibration modes is similar. The maximum relative error in resonance amplitude prediction by the 2D-ERM stands at 8.204%, highlighting the model’s capability to accurately predict the frequency-domain response of the panel. In contrast to the PMI damping layer, the T-SW308 damping layer significantly bolsters the panel’s damping efficiency while maintaining a relatively low stiffness level. An analysis of the time-domain data reveals the panel’s relatively low sensitivity to external excitations within the frequency band of 150∼300 Hz.
(2) An appreciable increase in the equivalent stiffness of the panel occurs with the elevation of the core layer height, where the rise in equivalent stiffness surpasses the increase in equivalent density, consequently leading to a notable enhancement in natural frequencies. Given that shear and torsional deformation modes significantly contribute to the vibration analysis, the overall structural vibration response is particularly responsive to alterations in shear and torsional stiffness. At a re-entrant angle of 40°, the sandwich panel demonstrates a heightened natural frequency and comparatively reduced resonance amplitude. Notably, adjustments in the aspect ratio exhibit minimal impact relative to other geometric parameters on the natural frequency.
(3) The hexagonal honeycomb sandwich panel and star-shaped honeycomb sandwich panel exhibit reduced sensitivity to external excitation. Furthermore, the star-shaped honeycomb sandwich panel not only displays lower sensitivity to external excitation but also features smaller displacement and velocity amplitudes during resonance. The 2D-ERM requires only a fraction of the time—1.9%, 2.6%, and 1.6%—compared to what the 3D-FEM necessitates for free-vibration and frequency- and time-domain analysis, respectively. Therefore, when dealing with three-dimensional spatial models that exhibit substantial geometric and physical complexity and necessitate a trade-off between model precision and computational efficiency, the VAM-based 2D equivalent model emerges as a more effective tool for dynamic analysis.

## Figures and Tables

**Table 1:** Material properties.

Material Parameter		Carbon Fiber	Epoxy Resin	Aluminium	PMI	T-SW308
Elasticity modulus	$E_{1}/\mathrm{GPa}$	105.5	4.5	70	263	430
	$E_{2}=E_{3}/\mathrm{GPa}$	11.3	4.5			
Shear modulus	$G_{12}=G_{13}/\mathrm{GPa}$	3.23	1.7	27	113	200
	$G_{23}/\mathrm{GPa}$	3.18	1.7			
Poisson’s ratio	$\nu_{12}=\nu_{13}$	0.28	0.38	0.3	-	-
	$\nu_{23}$	0.53	0.38			
Density	$\rho /g\cdot {\mathrm{cm}}^{-3}$	1.49	1.6	2.7	0.205	0.184

**Table 2:** Comparison of dynamic analysis efficiency between 2D-ERM and 3D-FEM.

Models	Element Type	Element Number	Node Number	Calculation Time (sec)		
				Free Vibration	Frequency-Domain Analysis	Time-Domain Analysis
Unit cell homogenization	C3D10	86,636	140,257	Homogenization time, 14		
2D-ERM	S4R	5400	5551	26	220	423
3D-FEM	C3D10	340,653	600,301	1362	8483	25,920

**Table 3:** Variation range of geometric parameters.

Geometrical Parameter				Layup Mode
$h_{c}/h_{f}$	$t_{c}/h_{c}$	α	$l_{4}/l_{2}$	
2.5∼15	0.2	$45^{\circ }$	1.5	[45/−45/0/90]s
10	0.05∼0.30	$45^{\circ }$	1.5	[45/−45/0/90]s
10	0.2	$10^{\circ }$∼$60^{\circ }$	1.5	[45/−45/0/90]s
10	0.2	$45^{\circ }$	1.1∼1.6	[45/−45/0/90]s

**Table 4:** Influence of geometric parameters on vibration characteristics of damped BHC-CSP.

Parameters	Ranges	|Δρ| $g/{\mathrm{cm}}^{3}$	$\left|\frac{\Delta A_{22}}{\Delta \rho }\right|$ $\mathrm{KN}\cdot {\mathrm{cm}}^{3}/g$	$\left|\frac{\Delta D_{22}}{\Delta \rho }\right|$ $\mathrm{KN}\cdot {\mathrm{cm}}^{4}/g$	$\left|\frac{\Delta \omega }{\Delta \rho }\right|$ $\mathrm{Hz}\cdot {\mathrm{cm}}^{3}/g$	$\left|\frac{\Delta R_{A}}{\Delta \rho }\right|$ ${\mathrm{cm}}^{4}/g$
$h_{c}/h_{f}$	2.5∼15	4.590	51.483	75.064	90.249	23.603
$t_{c}/h_{c}$	0.05∼0.30	4.286	120.885	10.174	50.246	0.446
α	$10^{\circ }∼60^{\circ }$	2.509	261.158	14.705	47.469	0.522
$l_{4}/l_{2}$	1.1∼1.6	0.874	13.365	8.423	31.732	0.499

## Data Availability

Data are available upon request due to restrictions, e.g., privacy or ethical. The data presented in this study are available upon request from the corresponding author. The data are not publicly available due to subsequent analyses and publications.

## References

[1] Sun G., Huo X., Wang H., Hazell P.J., Li Q. (2021). On the structural parameters of honeycomb-core sandwich panels against low-velocity impact. Compos. Part B-Eng..

[2] Ma Q., Rejab M.R.M., Siregar J.P., Guan Z. (2021). A review of the recent trends on core structures and impact response of sandwich panels. J. Compos. Mater..

[3] Qi C., Pei L.Z., Remennikov A., Yang S., Liu J., Wang J.S., Liao X.W. (2020). Parametric study and optimization of the protect system containing a re-entrant hexagon cored sandwich panel under blast impact. Compos. Struct..

[4] Chen G., Zhang P., Deng N., Cai S., Cheng Y., Liu J. (2022). Paper tube-guided blast response of sandwich panels with auxetic re-entrant and regular hexagonal honeycomb cores—An experimental study. Eng. Struct..

[5] Usta F., Türkmen H.S., Scarpa F. (2022). High-velocity impact resistance of doubly curved sandwich panels with re-entrant honeycomb and foam core. Int. J. Impact Eng..

[6] Ma N., Han Q., Han S., Li C. (2023). Hierarchical re-entrant honeycomb metamaterial for energy absorption and vibration insulation. Int. J. Mech. Sci..

[7] Razgordanisharahi A., Ghassabi A.A., Hellmich C. (2023). Free vibration analysis of cylindrical honeycomb sandwich panels using state-space Levy method. Thin-Walled Struct..

[8] Sun M., Wowk D., Mechefske C., Alexander E., Kim I.Y. (2022). Surface and honeycomb core damage in adhesively bonded aluminum sandwich panels subjected to low-velocity impact. Compos. Part B-Eng..

[9] Zhang X., Xu F., Zang Y., Feng W. (2020). Experimental and numerical investigation on damage behavior of honeycomb sandwich panel subjected to low-velocity impact. Compos. Struct..

[10] Li X., Zhang P., Wang Z., Wu G., Zhao L. (2014). Dynamic behavior of aluminum honeycomb sandwich panels under air blast, Experiment and numerical analysis. Compos. Struct..

[11] Lu Z.X., Li X., Yang Z.Y., Xie F. (2016). Novel structure with negative Poisson’s ratio and enhanced Young’s modulus. Compos. Struct..

[12] Chen Z., Wu X., Xie Y.M., Wang Z., Zhou S. (2020). Re-entrant auxetic lattices with enhanced stiffness, A numerical study. Int. J. Mech. Sci..

[13] Fu M.H., Chen Y., Hu L.L. (2017). A novel auxetic honeycomb with enhanced in-plane stiffness and buckling strength. Compos. Struct..

[14] Zhang X.Y., Ren X., Zhang Y., Xie Y.M. (2022). A novel auxetic metamaterial with enhanced mechanical properties and tunable auxeticity. Thin-Walled Struct..

[15] Yu R., Luo W., Yuan H., Liu J., He W., Yu Z. (2020). Experimental and numerical research on foam filled re-entrant cellular structure with negative Poisson’s ratio. Thin-Walled Struct..

[16] Luo H.C., Ren X., Zhang Y., Zhang X.Y., Zhang X.G., Luo C., Cheng X., Xie Y.M. (2022). Mechanical properties of foam-filled hexagonal and re-entrant honeycombs under uniaxial compression. Compos. Struct..

[17] Qi C., Jiang F., Remennikov A., Pei L.Z., Liu J., Wang J.S., Liao X.W., Yang S. (2020). Quasi-static crushing behavior of novel re-entrant circular auxetic honeycombs. Compos. Part B-Eng..

[18] Zhang X.C., An C.C., Shen Z.F., Wu H.X., Yang W.G., Bai J.P. (2020). Dynamic crushing responses of bio-inspired re-entrant auxetic honeycombs under in-plane impact loading. Mater. Today Commun..

[19] Saravanos D.A., Pereira J.M. (1992). Effects of interply damping layers on the dynamic characteristics ofcomposite plates. AIAA J..

[20] Wang H.J., Chen L.W. (2002). Vibration and damping analysis of a three-layered composite annular plate with a viscoelastic mid-layer. Compos. Struct..

[21] Yim J.H., Cho S.Y., Seo Y.J., Jang B.Z. (2003). A study on material damping of 0 laminated composite sandwich cantilever beams with a viscoelastic layer. Compos. Struct..

[22] Plagianakos T.S., Saravanos D.A. (2004). High-order layerwise mechanics and finite element for the damped dynamic characteristics of sandwich composite beams. Int. J. Solids Struct..

[23] Yang J.S., Xiong J., Ma L., Wang B., Zhang G., Wu L. (2013). Vibration and damping characteristics of hybrid carbon fiber composite pyramidal truss sandwich panels with viscoelastic layers. Compos. Struct..

[24] Berthelot J.M., Sefrani Y. (2006). Damping analysis of unidirectional glass fiber composites with interleaved viscoelastic layers, experimental investigation and discussion. J. Compos. Mater..

[25] Zhong Y.F., Qin W., Yu W. (2015). Variational asymptotic homogenization of magnetoelectro-elastic materials with coated fibers. Compos. Struc..

[26] Peng X., Zhong Y.F., Shi Z. (2021). Global buckling analysis of composite honeycomb sandwich plate with negative Poisson’s ratio (CSP-RHC) using variational asymptotic equivalent model. Compos. Struct..

[27] Chen J., Zhong Y., Luo Q., Shi Z. (2021). Static and dynamic analysis of Isogrid Stiffened Composite Plates (ISCP) using equivalent model based on variational asymptotic method. Thin-Walled Struct..

[28] Cesnik C.E., Hodges D.H. (1997). VABS, A new concept for composite rotor blade cross-sectional modeling. J. Am. Helicopter Soc..

[29] Peng X., Zhong Y.F., Shi Z. (2022). Free flexural vibration analysis of composite sandwich plate with reentrant honeycomb cores using homogenized plate model. J. Sound Vib..

[30] Grima J.N., Oliveri L., Attard D., Ellul B., Gatt R., Cicala G., Recca G. (2010). Hexagonal honeycombs with zero Poisson’s ratios and enhanced stiffness. Adv. Eng. Mater..

[31] Li H., Dong B., Cao J., Zhao J., Xiong J., Yang Y., Du D., Sun W., Wang X., Wu H. (2023). Vibration behaviours of foam-filled grille composite sandwich cylindrical shells. Int. J. Mech. Sci..

[32] Wattanasakulpong N., Eiadtrong S. (2023). Transient responses of sandwich plates with a functionally graded porous core, Jacobi–Ritz method. Int. J. Struct. Stab. Dyn..

